# Draft genome sequence of *Dongia* sp. strain agr-C8, isolated from the rhizosphere of *Phragmites australis* using a droplet-based cultivation method

**DOI:** 10.1128/mra.01011-25

**Published:** 2025-12-12

**Authors:** Tomoki Iwashita, Manabu Kanno, Yuri Ota, Satoko Matsukura, Masamune Morita, Akira Sasaki, Naohiro Noda, Tomoki Nishioka, Yuga Hirakata, Hideyuki Tamaki

**Affiliations:** 1 Biomanufacturing Process Research Center, National Institute of Advanced and Industrial Science and Technology (AIST) 73459, Tsukuba, Ibaraki, Japan; 2 Molecular Biosystems Research Institute, National Institute of Advanced and Industrial Science and Technology (AIST) 73459, Tsukuba, Ibaraki, Japan; Portland State University, Portland, Oregon, USA

**Keywords:** *Dongia*, *Phragmites australis*, droplet-based cultivation

## Abstract

We report the draft genome sequence of *Dongia* sp. strain agr-C8, isolated from the rhizosphere of *Phragmites australis* using a droplet-based cultivation method. The draft genome is 5,596,395 bp with a G+C content of 66.0%. The genome is predicted to contain 5,334 coding sequences and 3 rRNA genes.

## ANNOUNCEMENT

The genus *Dongia* was first described in 2010 (1) and includes five species. Members of this genus are found in soil, sediment, rhizosphere, and activated sludge (1–5). A recent study suggested that *Dongia* spp. may degrade polycyclic aromatic hydrocarbons, which are environmental pollutants (6). However, other physiological traits and ecological roles remain unclear. Here, we present the draft genome sequence of a new isolate, *Dongia* sp. strain agr-C8.

Microbial samples were collected from roots of *Phragmites australis* in Lake Kasumigaura, Tsuchiura City, Ibaraki, Japan (36.06° N, 140.22° E) on 12 October 2021. Root samples were homogenized using a previously described method (7). Homogenates were filtered through a 0.22 µm membrane filter (Merck, Germany), and the trapped microorganisms were suspended in 5 mL of 1/100-strength tryptic soy broth (Becton, Dickinson and Company, USA). Final cell concentration was adjusted to 7.1 × 10^7^ cells/mL using a hemocytometer (Sunlead Glass, Japan). Water-in-oil droplets were generated using a 2D chip-800DG (On-chip Biotechnologies, Japan) and an On-chip Droplet Generator (On-chip Biotechnologies), as described previously (8). Droplets were stored in 2 mL tubes and incubated at 25°C for 35 days. After incubation, droplets were dispensed into 96-well microplates containing 180 µL of 1/100-strength tryptic soy broth supplemented with 1.5% agar using the On-chip SPiS (On-chip Biotechnologies), based on a previous study (9). The microplates were incubated at 25°C for 28 days, and a single colony, designated strain agr-C8, was isolated. For genome sequencing, strain agr-C8 was cultured aerobically in R2A medium (10) at 25°C with shaking at 150 rpm for 5 days. Cells were harvested by centrifugation, and genomic DNA was extracted and fragmented using the Extrap Soil DNA Kit Plus Ver.2 (BDL, Japan), following the manufacturer’s protocol. DNA concentration was measured using a Qubit 3.0 Fluorometer (Thermo Fisher Scientific), and DNA quality was evaluated with a dsDNA 915 Reagent Kit (Agilent Technologies, USA). Whole-genome sequencing was performed using the DNBSEQ-T7 platform (MGI Tech). A short-read library was prepared from fragmented DNA using the MGIEasy Fast FS DNA Library Prep Set V2.0 (MGI Tech) and circularized with the MGIEasy Dual Barcode Circularization Kit (MGI Tech). Paired-end sequencing (2 × 150 bp) was performed using the DNBSEQ-T7 platform with a High-throughput Pair-End Sequencing Primer Kit (MGI Tech). Raw reads were filtered with fastp v0.23.2 (11) and assembled using SPAdes v3.13.0 (12). Genome annotation was performed using the DDBJ Fast Annotation and Submission Tool (DFAST) pipeline v1.3.4 (13) and BlastKOALA v3.1 (14). Genome completeness and contamination were assessed using CheckM v1.2.2 (15). All tools were run with default parameters unless otherwise specified. The genome assembly results are detailed in Table 1. The 16S rRNA gene sequence derived from the agr-C8 genome shared the highest similarity (95.63%) with *Dongia mobilis* CGMCC 1.7660^T^ (accession number NZ_SNYW01000000) in the EzBioCloud (16). Further genomic analysis revealed that this strain possesses all the genes related to assimilatory sulfur metabolism (*cysNC*, *cysH*, *cysI,* and *sseA* genes encoding sulfate adenylyltransferase subunit CysN, phosphoadenosine phosphosulfate reductase, sulfite reductase, and 3-mercaptopyruvate sulfurtransferase, respectively), which may contribute to the degradation of sulfur-containing pollutants.

**TABLE 1:** Overview of the draft genome sequence of strain agr-C8

Parameter	Value for agr-C8
Total no. of reads	2,268,122
Genome size (bp)	5,596,395
No. of contigs	8
G+C content (%)	66.0
N50 (bp)	1,384,326
No. of CDSs	5,334
No. of rRNA	3
No. of tRNA	53
Completeness (%)	100
Contamination (%)	0

## Data Availability

The draft genome sequence and annotations of *Dongia* sp. strain agr-C8 have been deposited in DDBJ/EMBL/GenBank under accession numbers BAAGHZ010000001, BAAGHZ010000002, BAAGHZ010000003, BAAGHZ010000004, BAAGHZ010000005, BAAGHZ010000006, BAAGHZ010000007, and BAAGHZ010000008. The genome sequence has also been submitted to the SRA under BioSample accession number SAMD00859344 and BioProject accession number PRJDB19910. Raw sequence data for strain agr-C8 were deposited under DRA accession number DRR633684.
